# A Pronounced Inflammatory Activity Characterizes the Early Fracture Healing Phase in Immunologically Restricted Patients

**DOI:** 10.3390/ijms18030583

**Published:** 2017-03-08

**Authors:** Paula Hoff, Timo Gaber, Cindy Strehl, Manuela Jakstadt, Holger Hoff, Katharina Schmidt-Bleek, Annemarie Lang, Eric Röhner, Dörte Huscher, Georg Matziolis, Gerd-Rüdiger Burmester, Gerhard Schmidmaier, Carsten Perka, Georg N. Duda, Frank Buttgereit

**Affiliations:** 1 Department of Rheumatology and Clinical Immunology, Charité University Hospital, 10117 Berlin, Germany; gaber@drfz.de (T.G.); strehl@drfz.de (C.S.); jakstadt@drfz.de (M.J.); annemarie.lang@charite.de (A.L.); huscher@drfz.de (D.H.); gerd.burmester@charite.de (G.-R.B.); frank.buttgereit@charite.de (F.B.); 2 German Rheumatism Research Center (DRFZ), 10117 Berlin, Germany; holger.hoff@icloud.com; 3 Department of Orthopedics, Jena University Hospital, Campus Eisenberg, 07607 Eisenberg, Germany; e.roehner@krankenhaus-eisenberg.de (E.R.); g.matziolis@krankenhaus-eisenberg.de (G.M.); 4 Endokrinologikum Berlin, 10117 Berlin, Germany; 5 Berlin-Brandenburg Center for Regenerative Therapies (BCRT), 13353 Berlin, Germany; carsten.perka@charite.de (C.P.); georg.duda@charite.de (G.N.D.); 6 Julius Wolff Institute, Charité University Hospital, 13353 Berlin, Germany; katharina.schmidt-bleek@charite.de; 7 Berlin-Brandenburg School for Regenerative Therapies (BSRT), 13353 Berlin, Germany; 8 Department of Orthopedics, University Hospital Heidelberg, 69118 Heidelberg, Germany; gerhard.schmidmaier@med.uni-heidelberg.de; 9 Center for Musculoskeletal Surgery, Charité University Hospital, 10117 Berlin, Germany

**Keywords:** immunologically restricted patients, fracture hematoma, inflammation, fracture healing, bone healing, cytokines, immune cells, chemokines

## Abstract

Immunologically restricted patients such as those with autoimmune diseases or malignancies often suffer from delayed or insufficient fracture healing. In human fracture hematomas and the surrounding bone marrow obtained from immunologically restricted patients, we analyzed the initial inflammatory phase on cellular and humoral level via flow cytometry and multiplex suspension array. Compared with controls, we demonstrated higher numbers of immune cells like monocytes/macrophages, natural killer T (NKT) cells, and activated T helper cells within the fracture hematomas and/or the surrounding bone marrow. Also, several pro-inflammatory cytokines such as Interleukin (IL)-6 and Tumor necrosis factor α (TNFα), chemokines (e.g., Eotaxin and RANTES), pro-angiogenic factors (e.g., IL-8 and Macrophage migration inhibitory factor: MIF), and regulatory cytokines (e.g., IL-10) were found at higher levels within the fracture hematomas and/or the surrounding bone marrow of immunologically restricted patients when compared to controls. We conclude here that the inflammatory activity on cellular and humoral levels at fracture sites of immunologically restricted patients considerably exceeds that of control patients. The initial inflammatory phase profoundly differs between these patient groups and is probably one of the reasons for prolonged or insufficient fracture healing often occurring within immunologically restricted patients.

## 1. Introduction

Patients suffering from disorders which impact their immune function often exhibit delayed or ineffective fracture healing [1,2,3,4,5,6,7,8,9], and sometimes even the development of pseudarthrosis [7,8,9]. Very heterogeneous circumstances are associated with restricted immune functions: inter alia autoimmune diseases, malignancies, diabetes mellitus, osteoporosis, and persons suffering from alcoholism, but also among the elderly [1,2,3,4,5,6]. The reasons for impaired fracture healing in these patients are not yet known in detail. Fracture healing is a complex regenerative process generally starting with inflammation [10]. After a trauma which leads to a fracture, a hematoma is formed in the fracture gap. This fracture hematoma represents the site of the initial inflammatory phase [11]. We characterized previously the initial inflammatory phase of control patients from the immunological point of view [12]. We could confirm the inflammatory nature of this initial phase both on RNA and protein levels showing high concentrations of pro-inflammatory cytokines such as IL-1β, Interferon (IFNγ or TNFα and chemokines like Monocyte chemotactic protein 1 (MCP-1), Interferon gamma-induced protein 10 (IP-10) and Regulated on activation, normal T cell expressed and secreted (RANTES) [12,13]. Furthermore, we demonstrated that immune cells invade and become activated [12]. Very little is known about these initial processes of fracture healing in patients with restricted immune functions. The heterogeneous group of immunologically restricted patients analyzed in this study is described in greater detail in the Section 4 “Materials and Methods” subheading Section 4.1 “Patients”. On the RNA level, a distinctly increased inflammation has already been described for immunologically restricted patients [14]. Furthermore, we have shown that immunologically restricted patients exhibit an inadequate response to bioenergetically adverse conditions like hypoxia which characterize the early milieu within the fracture gap [14]. Both enhanced inflammatory response and inadequate adaptation to hypoxia may lead to the decreased expression of Runt-related transcription factor 2 (RUNX-2) shown in the fracture hematomas of patients with restricted immune functions [14]. RUNX-2 is a transcription factor mediating osteogenesis [15,16,17].

Apart from this information, our knowledge about the initial inflammatory phase of fracture healing in immunologically restricted patients is still scarce and incomplete. This drove us to perform a detailed analysis of immune cell populations and pro-inflammatory and regulatory cytokines, chemokines, and factors regulating angiogenesis within fracture hematomas and the surrounding tissue in patients suffering from disorders concerning impact on immune functions. These results were then compared to those of previously analyzed data on fracture hematomas obtained from control patients (patients lacking co-morbidities associated with delayed fracture healing) [12]. Here, to our knowledge, we give the first detailed immunological characterization of the fracture hematoma from immunologically restricted patients.

## 2. Results

### 2.1. Fracture Hematomas of Immunologically Restricted Patients Comprise Higher Amounts of Monocytes/Macrophages, Hematopoietic Stem and Progenitor Cells, as Well as NKT Cells, While Less Regulatory T Cells Are Present, and T Helper Cells Exhibit an Activated Phenotype

**Figure 1:**
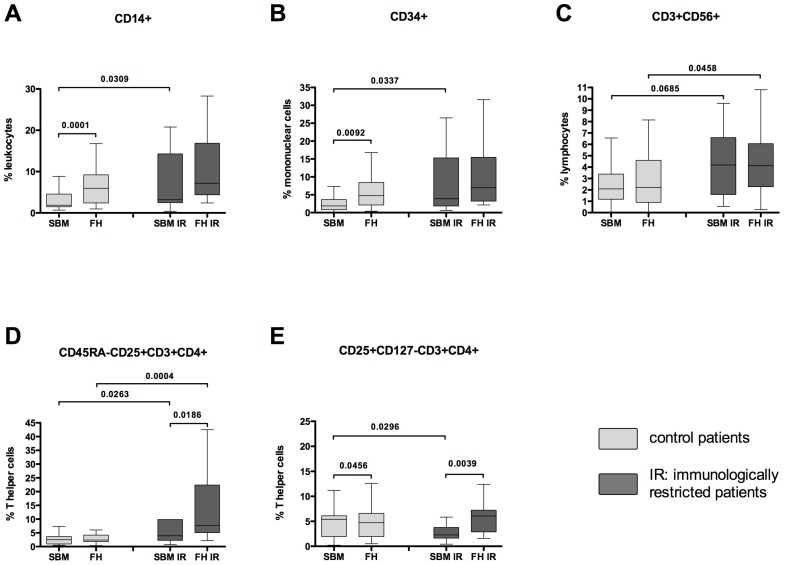
Monocytes/macrophages, hematopoietic stem and progenitor cells, natural killer T (NKT) cells and activated T helper cells accumulate in fracture hematoma (FH)/surrounding bone marrow (SBM) of immunologically restricted (FH IR/SBM IR) patients while regulatory T cells are decreased in SBM. Leukocytes from FH IR/SBM IR of immunologically restricted patients (dark grey) and controls (light grey) were isolated, stained with various surface markers and analyzed by flow cytometry. (**A**) Within all leukocytes, the CD14+ monocytes/macrophages were detected; (**B**) CD34+ hematopoietic stem and progenitor cells were detected within the mononuclear cells; (**C**) CD3+CD56+ NKT cells were detected within lymphocytes; (**D**) The activated CD45RA−CD25+CD3+CD4+ T helper cells were detected within the whole T helper cell population; (**E**) CD25+CD127−CD3+CD4+ regulatory T cells were detected within the whole T helper cell population. Controls *n* = 42, IR patients *n* = 20. IR-patients vs. controls Mann-Whitney *U* test, FH vs. corresponding SBM Wilcoxon test, *p*-values < 0.05 are shown as numbers in the Figure.

Immune cell populations were analyzed within the fracture hematoma (FH) and the surrounding bone marrow (SBM) of immunologically restricted (IR) patients (dark grey) and compared to FH and SBM of controls (light grey); the gating strategy is presented in Supplemental Figure 1. The amounts of CD14+ monocytes/macrophages (as percentage of all leucocytes found in the fracture hematoma) and CD34+ hematopoietic stem and progenitor cells (as percentage of all mononuclear cells) were increased significantly within SBM IR (surrounding bone marrow of immunologically restricted patients) when compared to SBM of controls and numerically within the FH IR when compared to FH of controls (Figure 1A,B). It should be noted that the data for controls presented in Figure 1 and elsewhere in this manuscript were taken from our recent publication [12]. The amounts of CD3+CD56+ natural killer T (NKT) cells (as percentage of all lymphocytes) and activated CD45RA−CD25+CD3+CD4+ T helper cells (as percentage of all T helper cells) were increased significantly within SBM IR (SBM of immunologically restricted patients) when compared to SBM of controls and significantly within the FH IR when compared to FH of controls (Figure 1C,D). The number of activated CD45RA−CD25+CD3+CD4+ T helper cells (as percentage of all T helper cells) within the FH IF was significantly increased when compared to SBM IR. The CD25+CD127−CD3+CD4+ regulatory T cells (as percentage of all T helper cells) were significantly decreased within SBM IR when compared to SBM, but were significantly increased within the FH IR when compared to SBM IR (Figure 1E). For the Mann-Whitney *U* test for independent groups, and Wilcoxon *t*-test for paired samples, statistically significant probability values of *p* < 0.05 are indicated.

### 2.2. Fracture Hematomas and the Surrounding Bone Marrow of Immunologically Restricted Patients Exhibit Higher Concentrations of Pro-Inflammatory Cytokines When Compared to Controls

**Figure 2:**
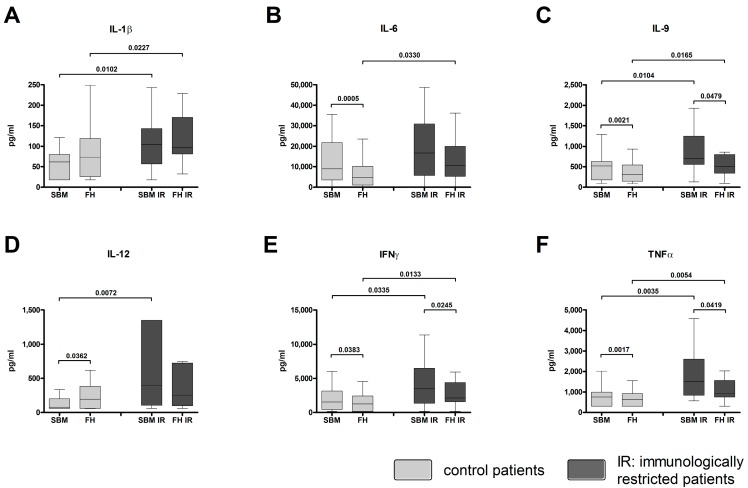
Pro-inflammatory cytokines are found at high concentrations in FH/SBM of immunologically restricted patients (FH IR/SBM IR). Supernatants from FH and SBM of immunologically restricted patients (FH IR/SBM IR: dark grey) and controls (FH/SBM: light grey) were analyzed for the concentrations of pro-inflammatory cytokines via multiplex suspension array. (**A**) Interleukin (IL)-1β; (**B**) IL-6; (**C**) IL-9; (**D**) IL-12; (**E**) Interferon γ (IFN)γ; (**F**) Tumor necrosis factor α (TNFα). Controls *n* = 42, IR patients *n* = 20. IR-patients vs. controls Mann-Whitney *U* test, FH vs. corresponding SBM Wilcoxon test, *p*-values < 0.05 are shown as numbers in the Figure.

Pro-inflammatory cytokines were quantified within the FH and SBM of controls (data previously published in [12]) and immunologically restricted (IR) patients. The concentrations of IL-1β, IL-9, IFNγ, and TNFα were significantly increased in both SBM IR and FH IR when compared to SBM and FH of controls (Figure 2A,C,E,F). The amount of IL-6 was significantly increased within the FH IR when compared to FH (Figure 2B). The concentration of IL-12 was significantly increased within the SBM IR when compared to SBM (Figure 2D). The amounts of IL-9, IFNγ, and TNFα were significantly decreased within the FH IR when compared to the corresponding SBM IR (Figure 2C,E,F). Mann-Whitney *U* test for independent groups, Wilcoxon *t*-test for paired samples, statistically significant probability values of *p* < 0.05 are indicated.

### 2.3. Regulatory Cytokines Are Increased at Fracture Sites of Immunologically Restricted Patients When Compared to Controls

**Figure 3:**
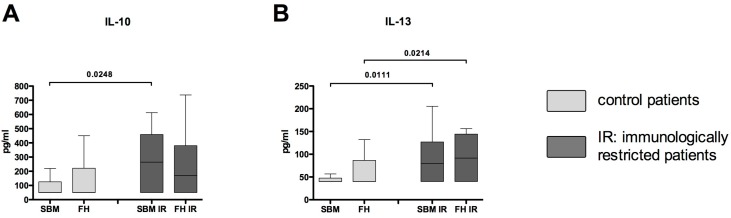
Regulatory cytokines are increased in FH/SBM of immunologically restricted patients. Supernatants from FH and SBM of immunologically restricted patients (FH IR/SBM IR: dark grey) and controls (FH/SBM: light grey) were analyzed for the concentrations of regulatory cytokines via multiplex suspension array. (**A**) IL-10; (**B**) IL-13. Controls *n* = 42, IR patients *n* = 20. IR-patients vs. controls Mann-Whitney *U* test, FH vs. corresponding SBM Wilcoxon test, *p*-values < 0.05 are shown as numbers in the Figure.

The concentration of IL-10 was significantly increased only within the SBM IR when compared to SBM (Figure 3A), while the concentration of IL-13 was significantly increased in both SBM IR and FH IR when compared to SBM and FH of controls (Figure 3B). For the Mann-Whitney *U* test for independent groups, and Wilcoxon *t*-test for paired samples, statistically significant probability values of *p* < 0.05 are indicated.

### 2.4. Fracture Hematomas and the Surrounding Bone Marrow of Immunologically Restricted Patients Exhibit Higher Concentrations of Chemokines When Compared to Controls

**Figure 4:**
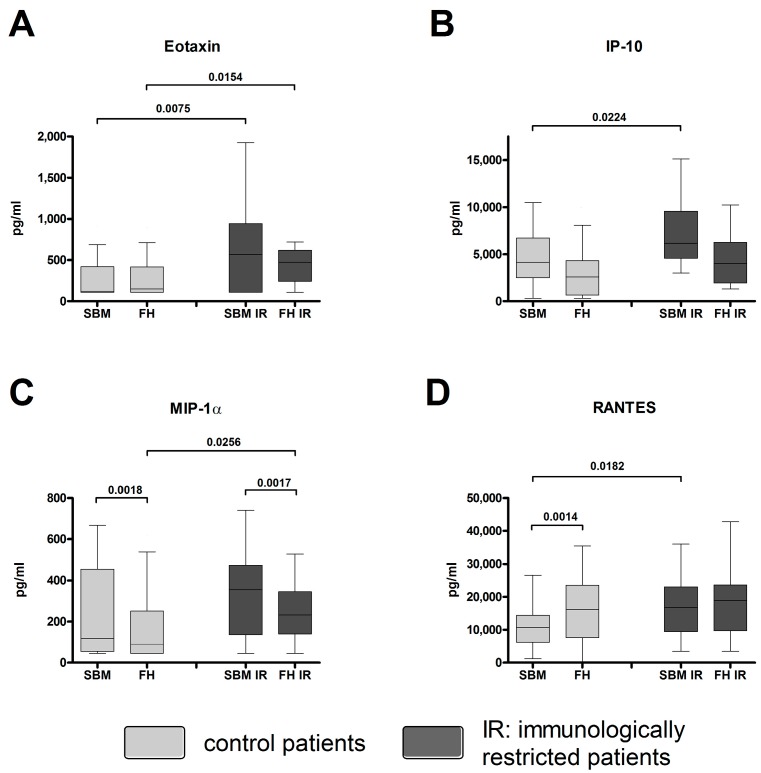
Chemokines are up-regulated in FH/SBM of immunologically restricted patients. Supernatants from FH and SBM of immunologically restricted patients (FH IR/SBM IR: dark grey) and controls (FH/SBM: light grey) were analyzed for the concentrations of chemokines via multiplex suspension array. (**A**) Eotaxin/CCL11; (**B**) Interferon gamma-induced protein 10 (IP-10/CXCL10); (**C**) Macrophage inflammatory protein 1α (MIP-1α/CCL3); (**D**) Regulated on activation, normal T cell expressed and secreted (RANTES/CCL5). Controls *n* = 42, IR patients *n* = 20. IR-patients vs. controls Mann-Whitney *U* test, FH vs. corresponding SBM Wilcoxon test, *p*-values < 0.05 are shown as numbers in the Figure.

Chemokines were quantified within FH and SBM of controls [12] and immunologically restricted (IR) patients. The concentration of Eotaxin was significantly increased in both SBM IR and FH IR when compared to SBM and FH of controls (Figure 4A). The concentrations of IP-10 and RANTES were significantly increased within the SBM IR when compared to SBM (Figure 4B,D). The amount of Macrophage inflammatory protein 1α (MIP-1α) was significantly increased within the FH IR when compared to FH (Figure 4C). The MIP-1α concentration was significantly increased within the SBM IR when compared to the corresponding FH IR (Figure 4C). For the Mann-Whitney *U* test for independent groups, and Wilcoxon *t*-test for paired samples, statistically significant probability values of *p* < 0.05 are indicated.

### 2.5. Factors Mediating Angiogenesis Were Found at Higher Concentrations within the Fracture Hematomas and the Surrounding Bone Marrow of Immunologically Restricted Patients When Compared to Controls

**Figure 5:**
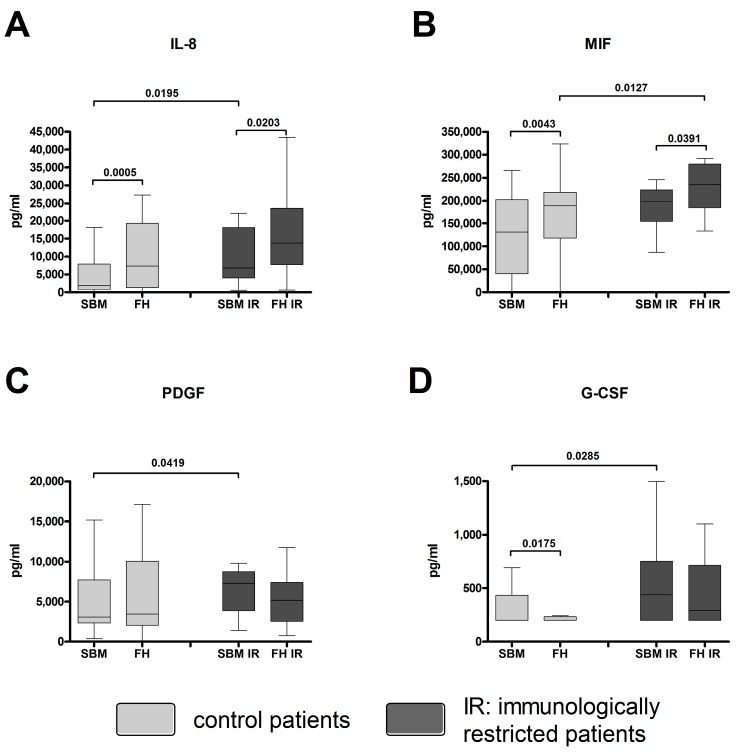
Angiogenic factors are highly secreted in FH/SBM of immunologically restricted patients. Supernatants from FH and SBM of immunologically restricted patients (FH IR/SBM IR: dark grey) and controls (FH/SBM: light grey) were analyzed for the concentrations of angiogenic factors via multiplex suspension array. (**A**) IL-8; (**B**) Macrophage migration inhibitory factor (MIF); (**C**) Platelet-derived growth factor (PDGF); (**D**) Granulocyte-colony stimulating factor (G-CSF). Controls *n* = 42, IR patients *n* = 20. IR-patients vs. controls Mann-Whitney *U* test, FH vs. corresponding SBM Wilcoxon test, *p*-values < 0.05 are shown as numbers in the Figure.

Angiogenic factors were quantified within the FH and SBM of controls [12] and immunologically restricted (IR) patients. The concentrations of IL-8, Platelet-derived growth factor (PDGF), and Granulocyte-colony stimulating factor (G-CSF) were significantly increased in SBM IR when compared to SBM of controls (Figure 5A,C,D). The amount of MIF was significantly increased within the FH IR when compared to FH (Figure 5B). The IL-8 and MIF concentrations were significantly increased within the FH IR when compared to the corresponding SBM IR (Figure 5A,B). For the Mann-Whitney *U* test for independent groups, and Wilcoxon *t*-test for paired samples, statistically significant probability values of *p* < 0.05 are indicated.

## 3. Discussion

The initial inflammatory phase of fracture healing differs between controls and immunologically restricted patients. As we have shown before, in controls (patients lacking co-morbidities associated with delayed fracture healing) there is significant inflammatory activity within the fracture hematoma (FH) and surrounding bone marrow (SBM) [12]. This situation was seen to be different to that found in immunologically restricted patients who are known to suffer more often from delayed or insufficient fracture healing [1,2,3,4,5,6,7,8,9], as shown in this work here. Previously, we could demonstrate pronounced inflammation and inadequate response to hypoxia in fracture hematomas of immunologically restricted patients on the RNA level [14]. Here we confirm these data on the protein level and add detailed information by extensively characterizing cellular composition, cellular activity, and cytokine/chemokine milieu.

### 3.1. Immune Cells in Fracture Hematoma of Immunologically Restricted Patients

We observed an increased invasion of monocytes/macrophages into the SBM of IR patients when compared to controls. It is well known that macrophages invade the fracture site and are essential for bone regeneration [18]. The higher number of them found in the IR-group is probably due to increased concentrations of chemokines like MIP-1α and RANTES (CCL3/5) which facilitate the immigration of monocytes/macrophages [19]. The overall increased inflammatory level within FH and SBM obtained from immunologically restricted patients presumably also leads to the high numbers observed for hematopoietic stem and progenitor cells, as well as NKT cells. The concentration of Eotaxin (CCL11) is high which could be responsible for the high number of CD34+ hematopoietic stem and progenitor cells (HSPC) which we saw [20]. In turn, monocytes, NKT cells but also HSPC become activated, proliferate, and then contribute to the inflammation via production of cytokines/chemokines. We could clearly demonstrate that the pronounced inflammatory situation within the fracture site in IR-patients leads to the activation of T helper cells as evidenced by the fact that CD25 is up-regulated, and CD45RA is down-regulated in this population. One possible explanation might be the relative lack of regulatory T cells as we could show lower numbers of CD25+CD127− regulatory T cells especially within the SBM of IR patients when compared to controls. We previously showed the activation of cytotoxic T cells at fracture sites in control patients [12]. Within the IR-group it seems to be the T helper cells which are being significantly more activated. This facilitates the production of pro-inflammatory cytokines, and we found these at higher concentrations within FH/SBM of the immunologically restricted patients when compared to controls.

### 3.2. Pro-Inflammatory and Regulatory Cytokines at the Fracture Site of Immunologically Restricted Patients

Also the concentration of IL-1β was significantly higher within the FH and SBM of immunologically restricted patients when compared to the controls. On the one hand, IL-1β contributes to the proliferation of osteoblasts and thus to bone regeneration [21,22], but on the other hand, a long exposure to IL-1β inhibits osteoblast migration and contributes to delayed healing [23]. We showed higher numbers of macrophages in the IR group which could be the source for the significantly increased concentrations of IL-6 and TNFα. IL-6 and TNFα together with the increased concentration of IL-1β could trigger increased osteoclastogenesis leading to increased resorption [24].

T helper cells (activated at fracture sites of IR-patients) produce IL-9 which is found at significantly higher concentrations in the IR-patients compared to controls, and this could lead to the observed higher number of hematopoietic stem and progenitor cells [25]. Mice lacking IL-12 and IL-23 show increased bone formation [26]. We demonstrated significantly higher concentrations of IL-12 within the immunologically restricted patients than those seen in the controls. IL-12 stimulates T helper cells to produce IFNγ which we have also shown to be significantly increased within the IR-patients. In turn, high IFNγ can inhibit the differentiation of mesenchymal stem cells and thus contribute to impaired fracture healing [26]. Mesenchymal stem cells are essential for fracture healing [27]. They migrate to the fracture site and are important progenitors of osteoblasts and bone lining cells [28]. However, they are also assumed to contribute to the termination of the initial inflammation in fracture healing via their immunomodulatory properties such as the secretion of IL-10 [29]. Thus, the significantly increased concentration of IL-10 could point to the higher activity of mesenchymal stem cells which might attempt to counter-regulate the profound inflammation in IR-patients. IL-13 is a regulatory cytokine closely related to IL-4 [30]. Activated T cells like those which we have shown to be present at fracture sites do secrete IL-13 and modulate monocyte and B cell function through IL-13 for example via the suppression of pro-inflammatory cytokine production [30,31]. At the fracture site we showed a significantly increased concentration of IL-13 which might be secreted in order to counter-regulate the elevated inflammation in the IR-patients.

### 3.3. Chemokines at Fracture Sites of Immunologically Restricted Patients

We showed an accumulation of monocytes/macrophages, NKT cells and activated T helper cells at fracture sites in immunologically restricted patients in comparison with the controls. These cells are able to secrete diverse chemokines but are also factors regulating vascularization [32,33,34,35]. Furthermore, the significantly increased concentration of Eotaxin probably explains the accumulation of CD34+ hematopoietic stem and progenitor cells within the immunologically restricted patients [20]. IP-10 might be up-regulated as a consequence of the increased concentration of IFNγ [36]. The increased amount of MIP-1α might contribute to disturbed osteoblast function, as MIP-1α is able to inhibit osteoblast differentiation [37]. RANTES is an important chemokine in bone homeostasis. It promotes chemo-attraction of osteoblasts and osteoblast survival [38]. Thus, RANTES is certainly important for bone regeneration and we also showed that secretion of RANTES in FH and SBM is present in the controls [12]. However, it is also osteoclasts which express the RANTES receptor CCR1 and thus the very high amount of RANTES might contribute to insufficient fracture healing in the immunologically restricted patients [38].

### 3.4. Angiogenic Factors in Fracture Hematomas of Immunologically Restricted Patients

The pronounced inflammatory activity within the FH/SBM of immunologically restricted patients is a high energy-consuming process [39]. Thus, the demand for revascularization is huge. Vascularization of course is essential for bone regeneration [40,41]. As we have shown before, there are high concentrations of factors mediating angiogenesis present at fracture sites of controls [12]. However, the pronounced inflammation within the immunologically restricted group might lead to an even significantly higher production of pro-angiogenic factors in IR-patients as we showed for IL-8, MIF, PDGF, and G-CSF.

## 4. Materials and Methods

### 4.1. Patients

We analyzed patients with closed fractures undergoing a surgery within 72 h post injury. All patients gave their written informed consent. The local ethical committee approved the study.

Patients meeting the inclusion and exclusion criteria summarized in Table 1 were defined as controls (*n* = 42) [12].

Patients with autoimmune diseases, cancer, diabetes mellitus, osteoporosis, or alcoholism were defined as immunologically restricted patients (*n* = 20) (see Table 2). These risk factors are known to be associated with prolonged or ineffective fracture healing [7,9,42,43,44,45]. Patients with the following autoimmune diseases were included: rheumatoid arthritis, cryoglobulinemic vasculitis, systemic lupus erythematosus, and giant cell arteritis. Patients with the following cancer diseases were included: non-Hodgkin lymphoma, 2 mamma carcinoma, and bronchial carcinoma.

### 4.2. Tissue Samples

Fracture hematoma (FH) and bone marrow surrounding the fracture hematoma (surrounding bone marrow, SBM): FH and SBM were obtained from patients (controls and immunologically restricted patients) with a closed fracture undergoing an osteosynthesis <72 h after fracture. The samples were kept in heparinized tubes to prevent coagulation of the SBM (Figure S1A), the FH was already coagulated when removed ex vivo. Thus, FH and SBM could be separated via filtration of the liquid SBM (70 µm cell strainer, BD Biosciences, Heidelberg, Germany) (Figure S1B) as described previously [12]. After separation of FH and SBM the coagulated FH was pressed through the cell strainer to prepare single cells (Figure S1C). All samples were centrifuged to separate cells and supernatant (Figure S1D). The cell-free supernatant was used for cytokine/chemokine analysis. The cell pellet was used for cytometric analysis.

Erythrocyte lysis was performed with cell pellets for 6 min at 4 °C (erythrocyte lysis buffer: 0.01 M KHCO_3_, 0.155 M NH_4_Cl, 0.1mM EDTA, pH 7.5). The samples were washed with phosphate buffered saline supplemented with 0.5% (*w*/*v*) bovine serum albumin (137 mM NaCl + 2.7 mM KCl + 1.5 mM KH_2_PO_4_ + 7.9 mM Na_2_HPO4·xH_2_O, pH 7.2 + 30 mM bovine serum albumin).

### 4.3. Flow Cytometry

Leukocytes were filtered (MACS pre-separation filter 30 µm, Miltenyi Biotech, Bergisch Gladbach, Germany) and incubated in a solution containing 5 mg/mL human IgG (IgG1 66.6%, IgG2 28.5%, IgG3 2.7% & IgG4 2.2%; Flebogamma, Grifols, Frankfurt, Germany) to block unspecific binding. Cells were stained with for 10 min at 4 °C with αCD3 (UCHT1), αCD56 (B159), αCD127 (hIL-7R-M21), αCCR7 (3D12), αCD4 (RPA-T4), αCD25 (M-A251), αCD19 (HIB19), αCD34 (581), αCD14 (M5E2), αCD16 (3G8), αCD69 (FN50), αIgD (IA6-2) (all from BD Biosciences, Heidelberg, Germany); αCD45RA (MEM-56) and αCD8 (3B5) (both from Caltag Laboratories, Hamburg, Germany) conjugated to Pacific blue, Pacific orange, PE-Cy7, PE, PE-Cy5, APC, APC-Cy7, APC-Alexa750 or FITC. Analysis was performed using a LSR II cytometer (BD Biosciences, Heidelberg, Germany) and FlowJo software (Tree Star, Ashland, OR, USA). Significantly different populations were presented when compared controls and immunologically restricted patients. Gating strategy is presented in Figure S2: granulocytes were defined according to scatter and via CD16 expression. Monocytes and macrophages were analyzed in scatter and via CD14 expression. Lymphocytes subpopulations were analyzed via expression of CD3, CD4, CD8, CD19, CD56, and their combinations. The activation and further differentiation was analyzed via the expression of CD25, CD69, CD45RA, CCR7, CD127, IgD. Only those results significantly different between controls and IR-patients are presented. Hematopoietic stem and progenitor cells were analyzed via the expression of CD34 within the mononuclear cell population defined in the scatter dot plot

### 4.4. Cytokines, Chemokines and Growth Factors

The concentrations of cytokines, chemokines and growth factors were measured by a Bioplex system (Bio-Rad Laboratories, Munich, Germany) according to the manufacturer’s instructions. The following cytokines and chemokines were quantified: interleukin (IL)-1β, IL-2, IL-5, IL-6, IL-7, IL-8, IL-9, IL-13, IL-15, IL-17, interferon-gamma (IFNγ), interferon-gamma-induced protein 10 (CXCL10, IP-10), tumor necrosis factor-alpha (TNFα), IL-1 receptor antagonist (IL-1ra), IL-4, IL-10, monocyte chemotactic protein-1 (MCP-1, CCL2), macrophage inflammatory protein 1α (MIP-1α, CCL3), MIP-1β (CCL4), Eotaxin (CCL11), basic fibroblast growth factor (FGF basic), platelet-derived growth factor (PDGF), vascular endothelial growth factor (VEGF), granulocyte colony-stimulating factor (G-CSF), granulocyte macrophage colony-stimulating factor (GM-CSF), CCL5 (regulated on activation normal T-cell expressed and secreted, RANTES), and MIF (macrophage migration inhibitory factor). Significantly different concentrations were presented when controls' and immunologically restricted patients' values were compared.

### 4.5. Statistical Analysis

Statistical tests were performed using Graph Pad Prism Software (Graph Pad, San Diego, CA, USA). Data are shown as box and whiskers using the Tukey method. Intra-individual differences between FH and the corresponding SBM were analyzed using the Wilcoxon *t*-test for paired samples. The differences between different patients groups (controls and immunologically restricted patients) were analyzed using the Mann-Whitney *U* test for independent groups. Probability values of *p* < 0.05 were considered to be statistically significant; the *p*-values are presented within the Figures.

## 5. Conclusions

**Figure 6:**
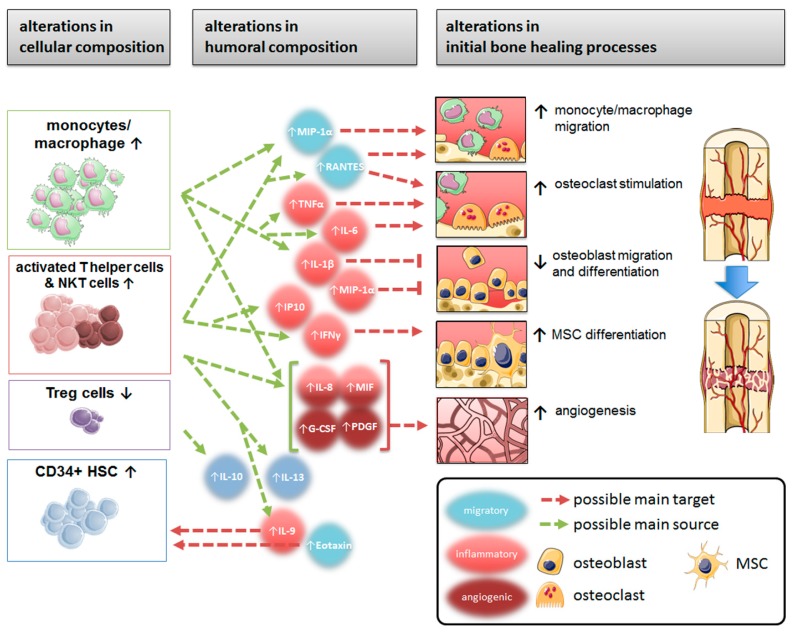
Summary of differences in FH/SBM of immunologically restricted patients when compared to control patients, and the influence on initial bone healing (green arrows connect suggested main sources to cytokines released; red arrows connect released cytokines to their suggested main target mechanisms and mode of action—↑up-regulation/↓down-regulation—in bone healing).

There is a pronounced inflammatory activity on cellular and humoral levels at the fracture site of immunologically restricted patients which significantly exceeds the normal inflammatory level of controls (Figure 6). The initial inflammatory phase differs between these patients and is probably one of the reasons for the prolonged or insufficient fracture healing often occurring within immunologically restricted patients.

## Figures and Tables

**Table 1:** Inclusion and exclusion criteria for patients defined as controls.

Inclusion Criteria	Exclusion Criteria (at Present or in the Past)
Patients with closed fractures Surgery within 72 h post injury	Autoimmune diseases
	Immunosuppressive drugs (such as MTX, glucocorticoids, cyclosporine, tacrolimus, sirolimus, biologics)
	Osteoporosis
	Bone metabolism-relevant drugs (such as bisphosphonates, glucocorticoids, denosumab, teriparatid)
	Chronic infections (e.g., HIV, HBV, HCV, Tbc)
	Cancer
	Diabetes mellitus
	Chronic kidney disease

**Table 2:** Inclusion and exclusion criteria for patients defined as immunologically restricted patients, the number of patients matching the inclusion criteria is given.

Inclusion Criteria	Exclusion Criteria (at Present or in the Past)
Patients with closed fractures	Chronic infections (such as HIV, HBV, HCV, Tbc)
Surgery within 72 h post injury	
AND one or more of the following conditions:	
Autoimmune diseases (*n* = 4)	
Osteoporosis (*n* = 6)	
Cancer (*n* = 4)	
Alcoholism (*n* = 4)	
Diabetes mellitus (*n* = 4)	
Chronic kidney disease (*n* = 3)	

The patient characteristics are given in Table 3.

**Table 3:** Patient characteristics.

Patients Groups	Years ± Standard Deviation	Age: Min–Max (years)	Male (%)	Female (%)
Healthy donors	53 ± 18.8	26–93	52.4	47.6
Immunologically restricted patients	70.1 ± 10.8	38–87	50	50

## Supplementary Materials

Supplementary materials can be found at www.mdpi.com/1422-0067/18/3/583/s1.
