# Supplementary material for: A Pronounced Inflammatory Activity Characterizes the Early Fracture Healing Phase in Immunologically Restricted Patients
Source: Int J Mol Sci. 2017 Mar 8;18(3):583. doi: 10.3390/ijms18030583 (PMC5372599; doi:10.3390/ijms18030583)
Supplement: Supplementary file 1 [file ijms-18-00583-s001.pdf]

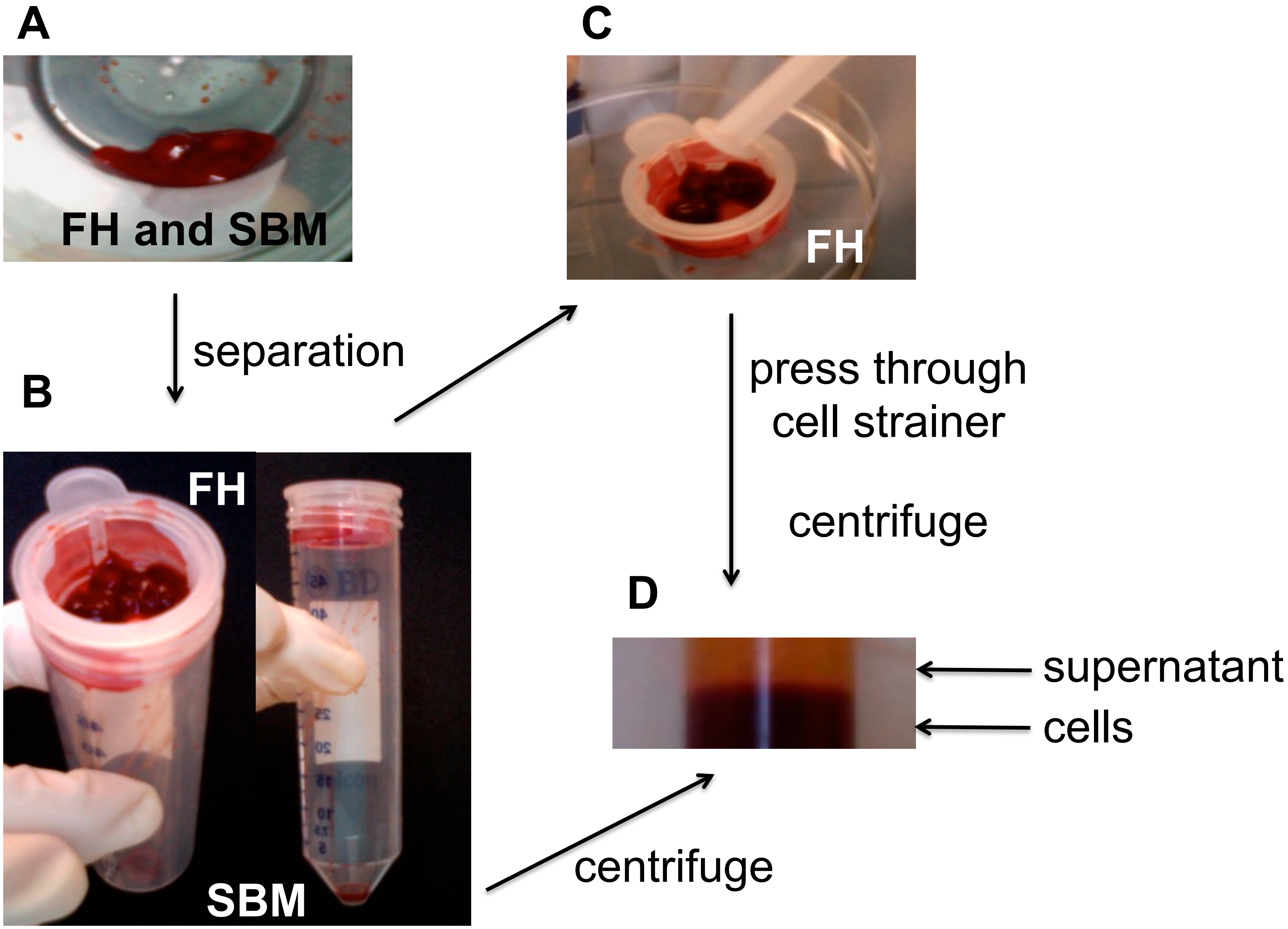
A
FH and SBM
separation
B
FH
SBM
C
FH
press through
cell strainer
centrifuge
D
supernatant
cells
centrifuge

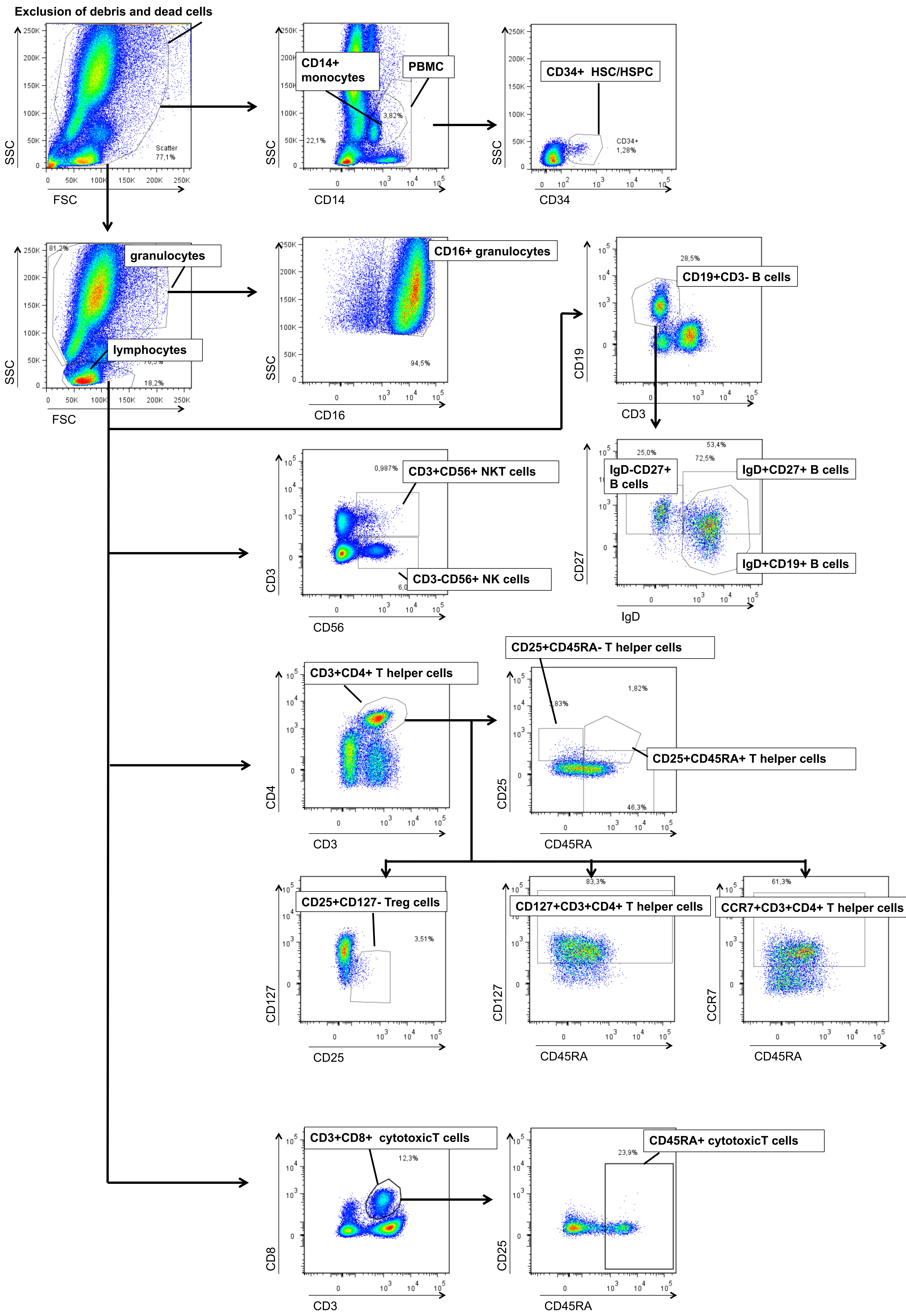

Exclusion of debris and dead cells
SSC
FSC
Scatter 77,1%
CD14+ monocytes
PBMC
CD14
22,1%
3,82%
CD34+ HSC/HSPC
CD34
CD34+ 1,28%
granulocytes
lymphocytes
81,2%
18,2%
CD16+ granulocytes
CD16
94,5%
CD19+CD3- B cells
CD19
CD3
28,5%
IgD-CD27+ B cells
IgD+CD27+ B cells
IgD+CD19+ B cells
CD27
IgD
25,0%
53,4%
72,5%
CD3+CD56+ NKT cells
CD3-CD56+ NK cells
CD56
0,987%
CD3+CD4+ T helper cells
CD4
CD25+CD45RA- T helper cells
CD25+CD45RA+ T helper cells
CD25
CD45RA
1,82%
46,3%
CD25+CD127- Treg cells
CD127
3,51%
CD127+CD3+CD4+ T helper cells
83,3%
CCR7+CD3+CD4+ T helper cells
CCR7
61,3%
CD3+CD8+ cytotoxicT cells
CD8
12,3%
CD45RA+ cytotoxicT cells
23,9%
